# Sensitivity, advantages, limitations, and clinical utility of targeted next-generation sequencing panels for the diagnosis of selected lysosomal storage disorders

**DOI:** 10.1590/1678-4685-GMB-2018-0092

**Published:** 2019-04-11

**Authors:** Diana Rojas Málaga, Ana Carolina Brusius-Facchin, Marina Siebert, Gabriela Pasqualim, Maria Luiza Saraiva-Pereira, Carolina F.M de Souza, Ida V.D. Schwartz, Ursula Matte, Roberto Giugliani

**Affiliations:** 1 Postgraduate Program in Genetics and Molecular Biology, Universidade Federal do Rio Grande do Sul, Porto Alegre, RS, Brazil.; 2 Medical Genetics Service, Hospital de Clinicas de Porto Alegre, Porto Alegre, RS, Brazil.; 3 Experimental Research Center, Hospital de Clinicas de Porto Alegre, Porto Alegre, RS, Brazil.; 4 Department of Genetics, Universidade Federal do Rio Grande do Sul, Porto Alegre, RS, Brazil.; 5 Gene Therapy Center, Hospital de Clínicas de Porto Alegre, Porto Alegre, RS, Brazil.; 6 Department of Biochemistry, Universidade Federal do Rio Grande do Sul, Porto Alegre, Brazil.

**Keywords:** Ion Torrent, molecular diagnostics, next-generation sequencing, lysosomal storage disorders, validation

## Abstract

Lysosomal storage disorders (LSDs) constitute a heterogeneous group of approximately 50 genetic disorders. LSDs diagnosis is challenging due to variability in phenotype penetrance, similar clinical manifestations, and a high allelic heterogeneity. A powerful tool for the diagnosis of the disease could reduce the “diagnostic odyssey” for affected families, leading to an appropriate genetic counseling and a better outcome for current therapies, since enzyme replacement therapies have been approved in Brazil for Gaucher, Fabry, and Pompe diseases, and are under development for Niemann-Pick Type B. However, application of next-generation sequencing (NGS) technology in the clinical diagnostic setting requires a previous validation phase. Here, we assessed the application of this technology as a fast, accurate, and cost-effective method to determine genetic diagnosis in selected LSDs. We have designed two panels for testing simultaneously 11 genes known to harbor casual mutations of LSDs. A cohort of 58 patients was used to validate those two panels, and the clinical utility of these gene panels was tested in four novel cases. We report the assessment of a NGS approach as a new tool in the diagnosis of LSDs in our service.

## Introduction

Lysosomal storage disorders (LSDs) comprise a heterogeneous group of at least 50 rare genetic disorders caused by progressive accumulation of specific substrates, generally due to a deficiency of a lysosomal enzyme (Filocamo and Morrone, 2011). A main factor related to diagnosis delay is the wide spectrum of clinical manifestations of variable severity that are not specific of the disorder and can overlap with symptoms of other LSDs (Vieira *et al.*, 2008; Martins *et al.*, 2013). Another challenge is the high allelic heterogeneity for genetic screening. Early diagnosis is important since enzyme replacement and other available therapies improve the natural course of many of these diseases (Tajima *et al.*, 2013; Muenzer, 2014; Franco *et al.*, 2016; Giugliani *et al.*, 2016).

The established approach to the diagnosis of patients with LSDs include the detection of the accumulated substrate whenever possible and the activity assay of the deficient enzyme, followed by Sanger sequencing of the gene associated with the disorder, which can be expensive and time consuming (Wang *et al.*, 2011). Fortunately, new technologies are becoming more accessible and relatively affordable for the diagnostic routine. Targeted next-generation sequencing (TNGS) allows the simultaneous screening of several LSDs-related genes, with great depth of coverage, manageable interpretation, and relative low risk of finding variants of unknown significance, decreasing turnaround times for the final report (Rehm *et al.*, 2013; Bhattacharjee *et al.*, 2015).

However, before using TNGS technologies as a diagnostic tool, the validation of each test offered in the clinical setting is required. This validation is essential for stablishing critical parameters, from sample processing to analysis and interpretation steps, following the recommendations of published guidelines (Gargis *et al.*, 2012; Rehm *et al.*, 2013).

Here, we present the development and validation of two different TNGS panels of genes related to a subgroup of LSDs, offered as a diagnostic alternative by a Brazilian reference service for rare diseases. The sensitivity, advantages, drawbacks, and clinical utility of these TNGS panels are then reported.

## Subjects and Methods

### Gene panel design

**Figure 1:**
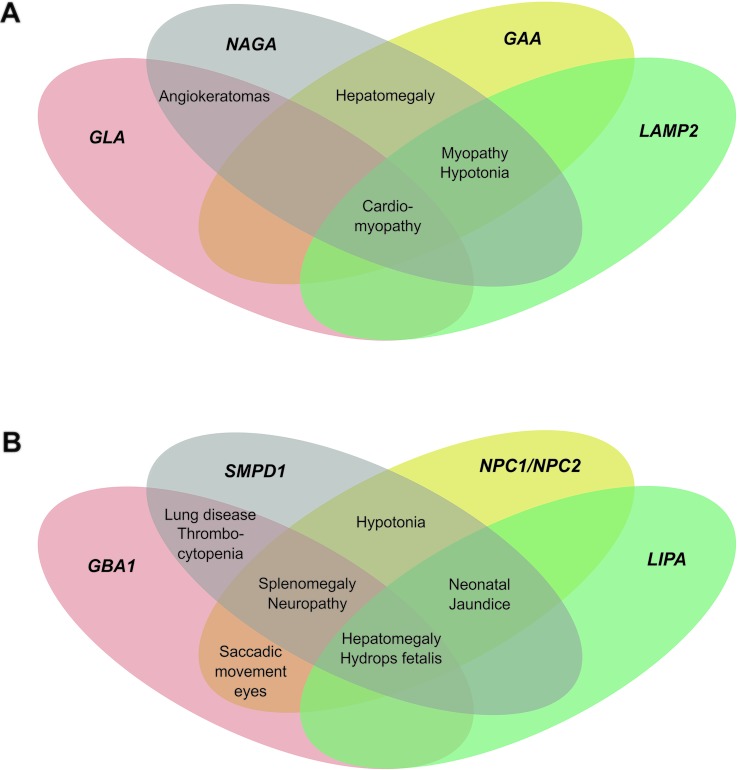
Overlapping of clinical manifestations among LSDs. Venn’s four-set diagram represented by causal genes. A, Panel A. B, Panel B.

Genes associated with LSDs with overlapping clinical manifestations, as well as related deficiencies were included in our panels (Figure 1). The two panels comprised 11 genes: Panel A: *GLA* (Fabry disease), *NAGA* (Schindler disease), *GAA* (Pompe disease), and *LAMP2* (Danon disease), and Panel B: *NPC1* (Niemann-Pick disease type C1), *NPC2* (Niemann-Pick disease type C2), *GBA1* (Gaucher disease)*, LIPA* (Lysosomal acid lipase deficiency)*, SMPD1* (Niemann-Pick disease type A/B), *CHIT1* (Chitotriosidase deficiency), and *PSAP* (Prosaposin deficiency and saposin B deficiency). Custom primers were designed using Ion Ampliseq^TM^ Designer v3.4 (Thermo Fisher Scientific) to generate a pool of primers for amplification of genomic regions of interest. Each one consists of two primer pools that target the entire coding region, including 20 bp of intron-exon junctions. Missed areas in the design were filled in by Sanger sequencing to reach a 100% breadth of coverage.

### Subjects

The validation phase was performed using whole blood genomic DNA extracted by a standard saline extraction method (Miller *et al.*, 1988), from 55 diagnosed patients (22 for panel A and 33 for panel B) who underwent previous investigation with biochemical tests and Sanger sequencing (with known mutations and polymorphisms, including SNPs and small *indels*). Samples from three healthy adults were also analyzed. All probands were recruited from patients attended at the Medical Genetic Service, Hospital de Clinicas de Porto Alegre, Brazil. All samples were anonymized, sequenced, and analyzed in a single blind manner. TNGS was performed using an Ion Torrent Personal Genome Machine^TM^ (PGM^TM^) System (Thermo Fisher Scientific). The clinical utility of the validated tests was assessed by evaluating four patients with suspected LSDs. The study was approved by the institutional Ethics Committee of HCPA (#15-0165).

### Multiplex PCR enrichment, library construction, and massive parallel sequencing

The reagents used in these analyses were from Thermo Fisher Scientific, unless otherwise stated. Twenty nanograms of each gDNA sample were used for PCR enrichment of targets by applying the two custom Ampliseq^TM^ panels. Each panel consisted of two separate PCR primer pools. The library was constructed using Ion AmpliSeq Library kit 2.0. Eight to nine samples barcoded with Ion Xpress Barcode Adapters kit were included in each set of library preparations. Unamplified libraries were purified with an Agencourt AMPure XP kit (Beckman Coulter). Libraries were prepared in equimolar concentrations using the Ion Library Equalizer kit, or quantified using the Qubit^®^ dsDNA HS kit, followed by dilution to the same concentration. For template preparation, the barcoded libraries were pooled in equimolar concentrations of 100 pM each and were subsequently submitted to emulsion PCR (emPCR) using the Ion PGM Template OT2 200 kit on the Ion OneTouch2 Instrument (Thermo Fisher Scientific). The percentage of positive Ion Sphere Particles (ISPs) was defined by flow cytometry performed on an Attune^®^ Acoustic Focusing Flow Cytometer (Thermo Fisher Scientific) according to the demonstrated protocol (Part. no. 4477181). Positive ISPs were enriched using Ion OneTouch ES (Enrichment System).

All barcoded samples were loaded onto Ion 314^TM^ chips v2 (Thermo Fisher Scientific) taking up to 8-9 samples on a single chip per sequencing run. Chip loading was performed according to the user guide for the Ion PGM sequencing 200 kit v2 (Thermo Fisher Scientific), following the manufacturer’s instruction.

### Data analysis

Raw signal data were analyzed using Torrent Suite Software v.5.0 (Thermo Fisher Scientific). Primary analyses included signal processing, base calling, demultiplexing, read alignment to human genome reference 19 (Genome Reference Consortium GRCh37), quality control of mapping quality, coverage analysis, and variant calling. Subsequently, a list of detected sequence variants, including SNPs and small insertions/deletions, was imported into Ion Reporter^TM^ Software (Thermo Fisher Scientific) for annotation. Alignments were visually verified with the Integrative Genomics Viewer (IGV) v2.3 (Robinson *et al.*, 2011).

Candidate variants met the following criteria: be detected on both strands and account for 20% of total reads at that site, quality score ≥ 20, minimum read depth of 100X and variant frequency in the population ≤ 1%. The filtered variants were then compared to mutation databases, including dbSNP (htpp://www.ncbi.nlm.nih.gov/projects/SNP/), 1000G (http://browser.1000genomes.org), ExAC (http://exac.broadinstitute.org), Online Archive of Brazilian Mutations (http://abraom.ib.usp.br/), HGMD (http://www.hgmd.cf.ac.uk/ac/), Pompe Center at Erasmus Medical Center (http://www.pompecenter.nl/), Fabry-database.org (http://fabry-database.org/), and the International Niemann-Pick Rare Disease Registry (https://inpdr.org/). All databases were last accessed in September 2017. Evaluation of the pathogenicity of the novel variants of unknown significance (VUS) (i.e., not found in any of the mutation databases, or not previously described in the literature) were analyzed with *in silico* web tools, such as SIFT (Kumar *et al.*, 2009; Sim *et al.*, 2012), Polyphen-2 (Adzhubei *et al.*, 2010), and Mutation Taster (Schwarz *et al.*, 2014), to predict potential protein deleterious effects on protein function. To evaluate the possible effect of synonymous variant in gene splicing, we used the Human Splicer Finding web tool (Desmet *et al.*, 2009). Indels were analyzed by VEST (Variant Effect Scoring Tool), VEP (Vep Effect Predictor), as well as Mutation Taster (Carter *et al.*, 2013; Douville *et al.*, 2016; McLaren *et al.*, 2016). Nonsense, frameshift, and canonical splice mutations were classified automatically as pathogenic (Richards *et al.*, 2015).

### Performance characteristics

Run metrics and coverage analyses were performed to identify systematic deficiencies. We analyzed depth of coverage (DoC) in the targeted amplicons to assess target enrichment across all 58 samples data sets and establish an acceptable reference range for key measures.

**Figure 2:**
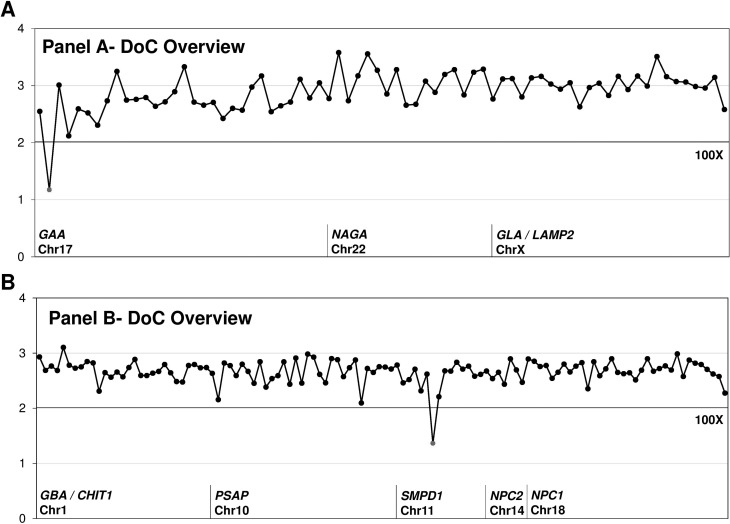
Depth of Coverage (DoC) of panels presented in this study. Overview of all 191 custom amplicons designed for Panel A (73 amplicons) (upper panel A) and Panel B (118 amplicons) (lower panel B) for TNGS. The line indicates a DoC of 100X. Relative DoC is on the y-axis and amplicons are on the x-axis.

Two coverage analyses were generated: (1) High-level DoC overview plot based on Tayoun *et al.* (2013), with relative DoC in the y-axis and amplicons on the x-axis, highlighting in red the amplicons with significantly lower coverage (Figure 2); and (2) relative DoC plot of exons per gene (Supplementary Figures S1 and S2). Direct visual inspection of amplicon reads on IGV v2.3 and evaluation of high-level DoC coverage overview were used to establish the reportable ranges for each panel.

Sensitivity and specificity were calculated (overall and for each gene) and compared with results obtained by standard Sanger sequencing. False negative and positive overall rates were also calculated. To assess reproducibility of the assay, we measured concordance between independent runs using relative DoC.

### Sanger sequencing

Sanger sequencing was performed for confirmation of all variants, to fill the regions missed by the custom panel design and low-coverage regions, and for the analysis of clinical utility. gDNA was amplified using specific primers designed for the free software Primer3 v.0.4.0. (available upon request). Amplicons were sequenced by both ends using the Big Dye Terminator v3.1 cycle sequencing kit (Thermo Fisher Scientific), and fragments were resolved on an ABI 3500 DNA Analyzer (Thermo Fisher Scientific). Analysis of results was performed with the BioEdit v7.2.5 free software.

## Results

### Run metrics

Our designs generated a total of 73 and 118 amplicons for Panel A and B, respectively. Mean amplicon read length was 150-180 bp. Sequencing of genes generated reads in the range of 69,000 to 7,600 per sample. An evenly distributed mean depth of coverage for both panels was achieved and a mean of 95% targeted bases were covered at least 100X. The other run metrics are summarized in Table 1.

**Table 1:** Performance characteristic for both LSDs panels

Panel	Breadth of coverage	Mapped reads per sample	On target	Mean depth (X)	Uniformity	% Target bases covered		
						20x	100x	500x
A	97.74%	76,729 ± 31195	0.95 ± 0.02	812 ± 339	0.91 ± 0.06	98.66 ± 0.49	95.55 ± 2.17	55.06 ± 18.08
B	99.67%	69,044 ± 26566	0.86 ± 0.05	498 ± 198	0.98 ± 0.01	99.72 ± 0.14	94.71 ± 6.41	56.42 ± 25.39

### Coverage analysis

An overview on coverage for all analyzed samples is shown in Figure 1. Although the coverage for *GAA* and *SMPD1* was expected to be 94.54 and 100%, respectively, the actual mean coverage was found to be 92.46 and 97.22%. The coverage analysis demonstrated two regions poorly covered in these genes, as shown in more detail in Figures S1 and S2. Unfortunately, the low covered region contained the location of the c.573delT (p.Ser192fs) *SMPD1* mutation. The actual coverage for all the other genes was as expected to be 100% based on probe design.

### Sensitivity

To assess the analytical sensitivity of the panels (Tables 2 and 3), we compared the results obtained by Sanger sequencing to those obtained by TNGS, including as many different types of variations as possible: nonsense, missense, small deletions, small insertions, splicing, and intronic variants. A total of 57 variants (pathogenic and polymorphisms) were analyzed (Table 4). We also identified their correct zygosity status (data not shown).

**Table 2:** Analytical sensitivity, specificity, FN and FP rates for both TNGS panels.

Panel	Pathogenic variants	Polymorphism NGS/Sanger	Sensitivity	Specificity	FN rate	FP rate
A	17/17	8/8	100% (25/25)*	99.96%	0.000%	0.040%
B	20/22	10/10	93.75% (30/32)**	99.97%	0.063%	0.033%

**Table 3:** Analytical sensitivity and specificity for each gene in our gene panels.

Panel	Gene	Sensitivity	Specificity
A	*GLA*	100% (10/10)	100% (1398/1398)
	*NAGA*	100% (3/3)	100% (1381/1381)
	*GAA*	100% (12/12)	99.89% (2987/2990)
	*LAMP2*	n.d*	100% (1707/1707)
B	*NPC1*	100% (8/8)	100% (4265/4265)
	*NPC2*	100% (1/1)	100% (5521/5521)
	*GBA1*	83.3% (5/6)	100% (1875/1875)
	*LIPA*	100% (4/4)	100% (1462/1462)
	*SMPD1*	91.7% (11/12)	99.8% (2775/2780)
	*CHIT1*	100% (1/1)	100% (1936/1936)
	*PSAP*	n.d*	100% (2184/2184)

Our assay identified precisely all recurrent mutations for LSDs, except two in Panel B. For this panel, the limitations were the inability to detect (1) *SMPD1* c.573delT, p.Ser192fs, located in a region with low coverage, and (2) *GBA1* c.[1448T > G; 1483G > C; 1497G > C], p.[Leu444Pro;Ala456Pro,Val460Val] (Tables 2 and 4).

**Table 4:** Variants detected in this study by TNGS and Sanger sequencing.

Gene	Sequence reference	Location	cDNA change	Protein change	dbSNP	Mutation type	NGS detected
*GLA*	NM_000169	Exon 01	c.32delG	p.Gly11fs	-	Deletion	Yes
		Exon 01	c.4C > T	p.Gln2Ter	-	Nonsense	Yes
		Exon 01	c.167G > A	p.Cys56Tyr	-	Missense	Yes
		Exon 02	c.334C > T	p.Arg112Cys	rs104894834	Missense	Yes
		Exon 03	c.456C > A	p.Tyr152Ter	-	Nonsense	Yes
		Exon 04	c.605G > A	p.Cys202Tyr	rs869312344	Missense	Yes
		Exon 05	c. 644A > G	p.Asn215Ser	rs28935197	Missense	Yes
		Exon 05	c.776C > G	p.Pro259Arg	-	Missense	Yes
		Exon 05	c. 790G > T	p.Asp264Tyr	rs190347120	Missense	Yes
		Exon 07	c.1102G > A	p.Ala368Thr	rs144994244	Missense	Yes
*NAGA*	NM_000262.2	Exon 03	c. 279G > A	p.Pro93Pro	rs133369	Missense	Yes
		Exon 06	c.720G > A	p.Gln240Gln	-	Missense	Yes
		Exon 08	c.973G > A	p.Glu325Lys	rs121434529	Missense	Yes
*GAA*	NM_001079804	Intron 01	c.-32-13T > G	-	rs386834236	Splicing	Yes
		Exon 03	c.596A > G	p.His199Arg	rs1042393	Missense	Yes
		Exon 03	c.668G > A	p.Arg223His	rs1042395	Missense	Yes
		Intron 8	c.1327-18A > G	-	rs2278619	Intron variant	Yes
		Exon 09	c.1374C > T	p.Tyr458Tyr	rs1800305	Missense	Yes
		Exon 10	c.1465G > A	p.Asp489Asn	rs398123169	Missense	Yes
		Exon 10	c.1504A > G	p.Met502Val	rs376067362	Missense	Yes
		Exon 14	c.1905C > A	p.Asn635Lys	-	Missense	Yes
		Exon 14	c.1941C > G	p.Cys647Trp	-	Missense	Yes
		Intron 14	c.2040+20A > G	-	rs2304836	Intron variant	Yes
		Exon 15	c.2065G > A	p.Glu689Lys	rs1800309	Missense	Yes
		Exon 18	c.2560C > T	p.Arg854Ter	rs121907943	Nonsense	Yes
*NPC1*	NM_000271.4	Exon 02	c.114_122del GAGGTACAA	p.Lys38_Tyr40del	-	Deletion	Yes
		Exon 05	c.530G > A	p.Cys177Tyr	rs80358252	Missense	Yes
		Exon 5,∐8,∐12	c.[547G > A;1093T > C;1937G > A]	p.[Ala183Thr;Ser365Pro;Arg646His]	rs111256741,∐-,∐ rs112387560	Missense	Yes
		Exon 20	c.3019C > G	p.Pro1007Ala	rs80358257	Missense	Yes
		Exon 21	c.3104C > T	p.Ala1035Val	rs28942107	Missense	Yes
		Exon 21	c.3182T > C	p.Ile1061Thr	rs80358259	Missense	Yes
		Intron 22	c.3477+3 insCA	-	-	Insertion	Yes+
		Exon 24	c.3662_3662delT	p.Phe1211fs	-	Deletion	Yes
*NPC2*	NM_006432	Exon 01	c.58G > T	p.Glu20Ter	rs80358260	Nonsense	Yes
*GBA1*	NM_001005742	Exon 07	c.850C > A	p.Pro245Thr	-	Missense	Yes
		Exon 07	c.982_983insTGC	p.Leu327dup	rs121908298		Yes
		Exon 09	c.1226A > G	p.Asn370Ser	rs76763715	Missense	Yes
		Exon 09	c.1251G > C	p.Trp378Cys	-	Missense	Yes
		Exon 10	c.1448T > G	p.Leu444Pro	rs421016	Missense	Yes
		Exon 10	c.[1448T > G;1483G > C;1497G > C]	p.[Leu444Pro; Ala456Pro; Val460Val]	-	Missense	No
*LIPA*	NM_001127605	Exon 02	c.67G > A	p.Gly23Arg	rs1051339	Missense	Yes
		Exon 08	c.894G > A	p.Glu298Glu	rs116928232	Missense	Yes
		Exon 10	c.1204G > A	p.Gly342Arg	-	Missense	Yes
		Intron 05	c.539-5C > T	-	rs2297472	Intron variant	Yes
*SMPD1*	NM_000543	Exon 01	c.107T > C	p.Val36Val	rs1050228	Missense	Yes
		Exon 02	c.338G > A	p.Arg113His	rs149770879	Missense	Yes
		Exon 02	c.573delT	p.Ser192fs	rs727504167	Deletion	No
		Exon 02	c.636T > C	p.Asp212Asp	rs7951904	Missense	Yes
		Exon 02	c.690C > G	p.Arg230Arg	-	Missense	Yes
		Exon 02	c.714A > G	p.Ala238Ala	rs2682091	Missense	Yes
		Exon 02	c.739G > A	p.Gly247Ser	rs587779408	Missense	Yes
		Exon 06	c.1522GC	p.Gly508Arg	rs1050239	Missense	Yes
		Exon 06	c.1749G > A	p.Ser583Ser	rs35098198	Missense	Yes
		Exon 06	c.1805G > C	p.Arg602Pro	-	Missense	Yes
		Exon 06	c.1805G > A	p.Arg602His	rs370129081	Missense	Yes
		Exon 06	c.1826_1828delGCC	p.Arg608del	rs120074118	Deletion	Yes
*CHIT1*	NM_003465.2	Exon 04	c.304G > A	p.Gly102Ser	rs2297950	Missense	Yes

### Specificity

Of all sequenced DNA samples, we identified threee false positives in panel A (3 in 7476 true negatives) and five false positives in panel B (5 in 15,054 true negatives), resulting in a specificity value of 99.96% (95%CI=0.998-0.999) and 99.97% (95%CI=0.9992-0.999), respectively (Table 2). Specificity by gene is shown in Table 3. These false positives were located in low coverage regions, which are prone to sequencing errors.

### Reproducibility

**Figure 3:**
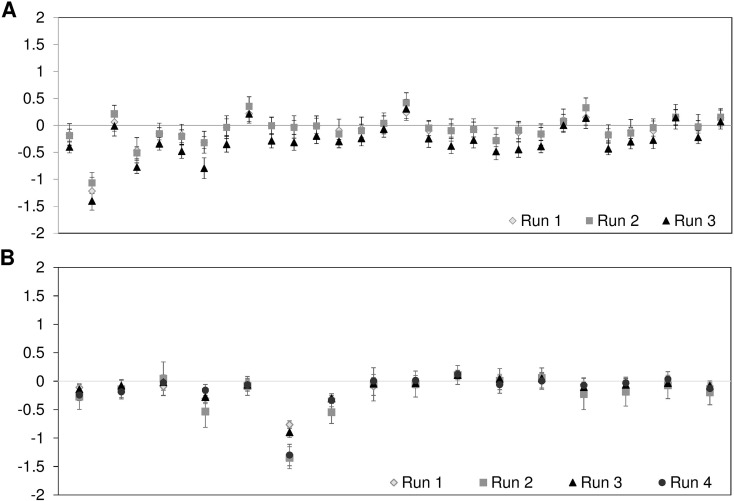
Reproducibility of assays. (A) Mean relative DoC at 30 amplicons (*GAA* gene) of 8 different samples sequenced in 3 different assay runs; (B) Mean relative DoC at 16 amplicons (*SMPD1* gene) of 8-9 different samples sequenced in 4 different assay runs. Error bars represent standard deviation.

To determine the reproducibility of our assay, we sequenced 24 samples divided in three independent runs for Panel A, and 34 samples divided in four independent runs for Panel B (Figure 3).

### Clinical Utility Assessment

#### Case 1

A 15-year-old male patient with suspicion of having a LSD was referred to our service via NPC Brazil Network. The main clinical findings were unexplained hepatosplenomegaly and myelogram with presence of numerous histiocytes. Several biochemical assays were performed to reach a diagnosis, including measurement of oxysterol and activity of chitotriosidase, lysosomal acid lipase, and b-galactosidase as reference enzymes that were all within normal ranges. Filipin test was inconclusive. Eventually, NPA/B was suspected and ASM enzyme activity was tested in cultured skin fibroblasts, resulting in 1.25 nmol/h/mg protein (reference value: 49-72), indicating NPA/B disease. Due to several factors, like request of new samples for the biochemical assays, it took approximately 12 months to reach this biochemical diagnosis. Panel B, which includes genes related to LSD with hepatosplenomegaly as common clinical manifestation, was utilized as second-tier diagnostic approach. We found two pathogenic variants in *SMPD1*, both confirmed by Sanger sequencing: p.Arg610del (c.1826_1828delGCC) (rs120074118) and p.Asp420fs (c.1259delA), the latter being a novel, unreported mutation and not found in controls (n=32).

#### Case 2

A 21-month-old patient, daughter of consanguineous parents who presented macrocephaly and hepatosplenomegaly as main clinical features, high cholesterol (228 mg/dL) and triglycerides (492 mg/dL) levels, elevated liver enzymes (GGT: 137 IU/L; TGP: 256 IU/L) as well as low levels of sphingomyelinase activity, was referred for molecular analysis of *SMPD1* gene. TNGS (Panel B) revealed the homozygous small deletion p.Leu474fs (c.1420_1421delCT), which was reported previously as being pathogenic (rs398123476).

#### Case 3

A 21-year-old female, child of a non-consanguineous marriage, with diagnosis of hypertrophic cardiomyopathy at 18 years of age and with previous diagnosis of Danon disease, was referred to our service for mutation analysis of the *LAMP2* gene. TNGS (Panel A) detected the hemizygous variant p.Asn242fs (c. 725delA), a novel pathogenic variant.

#### Case 4

A 16-year-old male with suspicion of Danon disease due to hypertrophic cardiomyopathy with anomalous pathway, presented intellectual deficiency, proximal myopathy, and alterations in liver tests. As in case 3, Panel A was used, detecting the hemizygous variant c.741+1G > A, described as pathogenic (HGMD CS003703).

## Discussion

Due to various reasons, such as wide clinical and genetic heterogeneity, LSDs are difficult to diagnose, and it can take several years to reach a final diagnosis (Vieira *et al.*, 2008; Martins *et al.*, 2013). Even if no treatment is available for many of these disorders, genetic diagnosis has potential benefits, such as predicting the prognosis,and allowing genetic counselling and family screening. Recent studies highlight the clinical utility of TNGS technology for genetic diagnosis of LSDs (Wood *et al.*, 2013; Fernandez-Marmiesse *et al.*, 2014; Lévesque *et al.*, 2016). Although there are some drawbacks to TNGS, such as the inability to detect large indels and structural variants, there are several advantages in applying this approach early in the investigation of patients with LSDs, like high coverage, completeness, low rate of incidental finding, and the potential to reduce the diagnostic delay. TNGS assays involve various technical steps, starting from sample preparation to analysis and data interpretation, and each one requires full validation. We presented data on the development and validation of two gene panels, designed following the criteria of overlapping clinical manifestations, to be offered as a diagnostics option by a reference center of rare diseases (Figure 1). Prior to TNGS, an enrichment step of the genes included in the panel is necessary through capture approaches based on hybridization or PCR-based strategies. The latter one is especially suitable for the investigation of regions with less than 100 kb, has versatile design, and is the most convenient for analysis of genes with pseudogenes due to its high specificity, sensitivity, and reproducibility (Claes and De Leener, 2014). Despite some reported disadvantages for this approach (time-consuming, uneven coverage of the target regions due to unequal PCR efficiency across the various amplicons, allelic dropout, and difficulties to detect large deletion/insertion events), Ion Ampliseq targeted technology utilizes a PCR-based method (high throughput multiplex PCR) for this purpose, overcoming some of the limitations and providing high specificity (here, 99.96% and 99.97%) and uniformity (91%-98% for both panels).

From the run metrics results, we can conclude that all samples were uniformly covered at depths that exceed the minimum coverage required (100 X) for accurate calling of variants. The bioinformatics pipeline applied here demonstrated high sensitivity for Panel A (sensitivity 100%) and Panel B (sensitivity 93.75%) (Table 1). The use of normal controls (n=3) allowed the identification of eight platform-specific false positive variants, which were filtered from subsequent analyses. A high reproducibility was observed revealing a high concordance between independent runs.

Inadequate coverage regions were identified by coverage plots (Figure 2), and these regions werfe completed by Sanger sequencing. A technical difficulty encountered was related to enrichment of some targets and, as a consequence, low sequence coverage was found. This was observed at two targets corresponding to the *GAA* and *SMPD1* genes. A high GC-content region (70%) was probably the main reason why the GAA amplicon was poorly covered (~20X). For *SMPD1*, a low-covered amplicon (~30X) was identified, showing both a GC-content of 66% and a homopolymeric region within the target. These are well-recognized limitations of NGS sequencing. As recommended by the American College of Medical Genetics, both tests achieved a 100% breadth of coverage when complemented with gold-standard DNA sequencing that improves clinical sensitivity.

Another major limitation of Panel B was the inability to detect the *RecNci*I allele, c.[1448T > G; 1483G > C; 1497G > C]/ p.[Leu444Pro;Ala456Pro,Val460Val]. High sequence similarity between functional genes and their pseudogenes can make the detection of genuine mutation difficult due to the ambivalent mapping in the analysis of NGS data, which cannot always be avoided. Sanger sequencing is generally used to elucidate the correct variant mapping (Claes and De Leener, 2014). In our study, the presence of *GBAP1*, a highly homologous *GBA1* pseudogene, complicated sequencing analysis by NGS, with the *RecNci*I allele being particularly difficult to assess since mutant bases in *GBA1* (exon 10) are the wild type sequence in its pseudogene. Panel B failed to detect this allele, representing a case of false negative when present, because variant-containing reads align to homologous loci. Our strategy to infer the presence of *RecNci*I was based on the employment of a global alignment strategy, analyzing the DoC of exon 10 *GBA1* and the homologous *GBAP1* region. We observed that in the presence of the *Rec* allele an uneven reads distribution was observed due to the exclusive alignment of variant-containing reads with *GBAP1*. As examples: (a) for homozygous N370S, we observed a DoC of 369 X for *GBA1* exon 10 and 370X for *GBAP1* homologous region; (b) in the case of compound heterozygosity (N370S/*RecNci*I) for exon 10 *GBA1*, a DoC of 144X and 313X for *GBAP1* was observed. Therefore, the presence of this *Rec* allele was inferred, but Sanger sequencing using a specific primer pair for exon 10 was required for confirmation of this inference.

Clinical utility assessment was performed. Two pathogenic variants were found, *SMPD1* p.Asp420fs (c.1259delA) and *LAMP2* p.Asn242fs (c. 725delA), demonstrating that our TNGS panel is a sensitive tool, with faster turnaround times for provision of results, and relative low cost (~USD 320 per sample) when compared with Sanger sequencing of individual genes, and showing the potential role for diagnosis of LSDs in our Medical Genetics Service.

In conclusion, TNGS technology can be used for the simultaneous testing of a broad range of SNPs and indels, being a fast, accurate, and cost effective method for the diagnosis of selected LSDs. It allows faster diagnosis and earlier treatment of patients, contributing to reduce the morbidity of the diseases and improve patient survival and quality of life.

## Supplementary material

The following online material is available for this article:

Figure S1 Relative Depth of Coverage, Panel A.

Figure S2 Relative Depth of Coverage, Panel B.

*Associate Editor: Mariluce Riegel*
