# Supplementary material for: Sensitivity, advantages, limitations, and clinical utility of targeted next-generation sequencing panels for the diagnosis of selected lysosomal storage disorders
Source: Genet Mol Biol. 2019 Apr 11;42(1 Suppl 1):197–206. doi: 10.1590/1678-4685-GMB-2018-0092 (PMC6687342; doi:10.1590/1678-4685-GMB-2018-0092)
Supplement: Supplementary file 1 [file 1415-4757-GMB-1678-4685-GMB-2018-0092-20190318-suppl1.pdf]

Supplementary Material to "Sensitivity, advantages, limitations, and clinical utility of targeted next-generation sequencing panels for the diagnosis of selected lysosomal storage disorders"

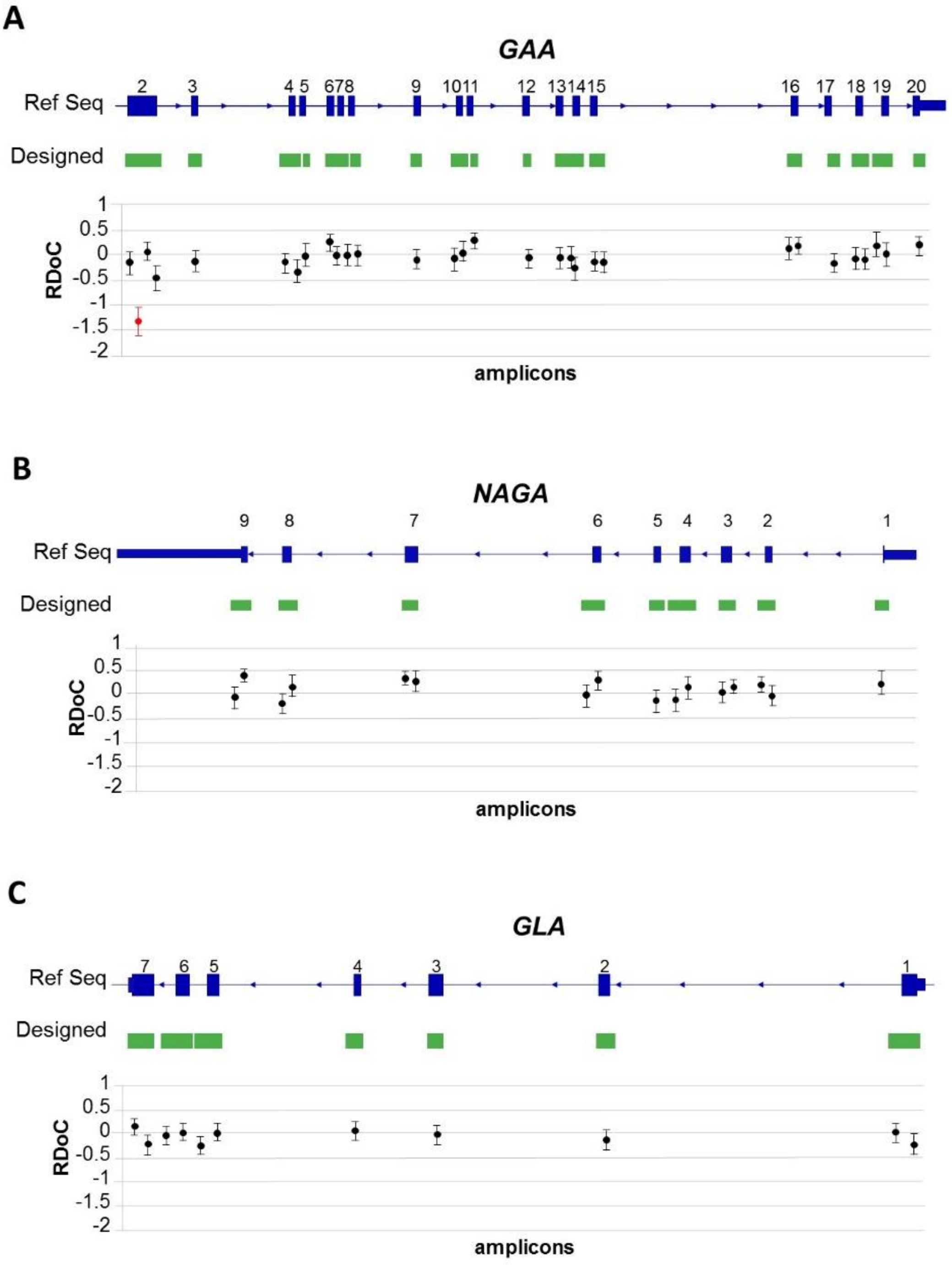

D

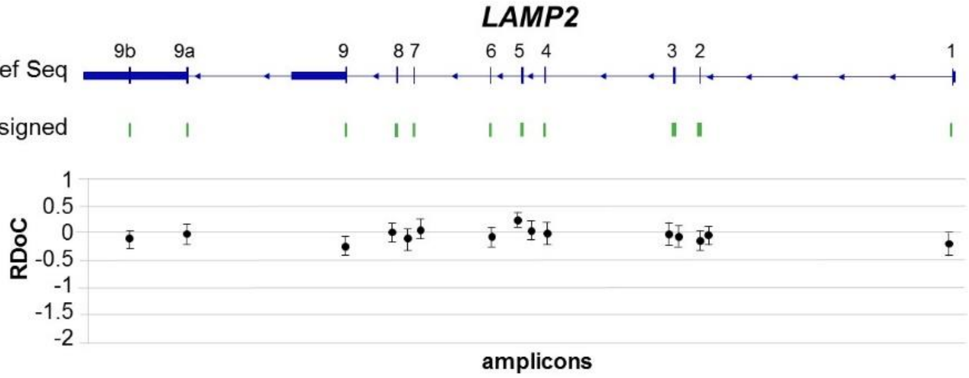

Figure S1 - Relative Depth of Coverage (RDoC) of 73 custom amplicons corresponding to eight-nine samples analyzed in one run. In blue is represented the reference sequence indicating the coding exons and in green the targets designed for NGS sequencing. A) *GAA*, B) *NAGA*, C) *GLA* and D) *LAMP2*.
