# Supplementary material for: Sensitivity, advantages, limitations, and clinical utility of targeted next-generation sequencing panels for the diagnosis of selected lysosomal storage disorders
Source: Genet Mol Biol. 2019 Apr 11;42(1 Suppl 1):197–206. doi: 10.1590/1678-4685-GMB-2018-0092 (PMC6687342; doi:10.1590/1678-4685-GMB-2018-0092)
Supplement: Supplementary file 2 [file 1415-4757-GMB-1678-4685-GMB-2018-0092-20190318-suppl2.pdf]

Supplementary Material to "Sensitivity, advantages, limitations, and clinical utility of targeted next-generation sequencing panels for the diagnosis of selected lysosomal storage disorders"

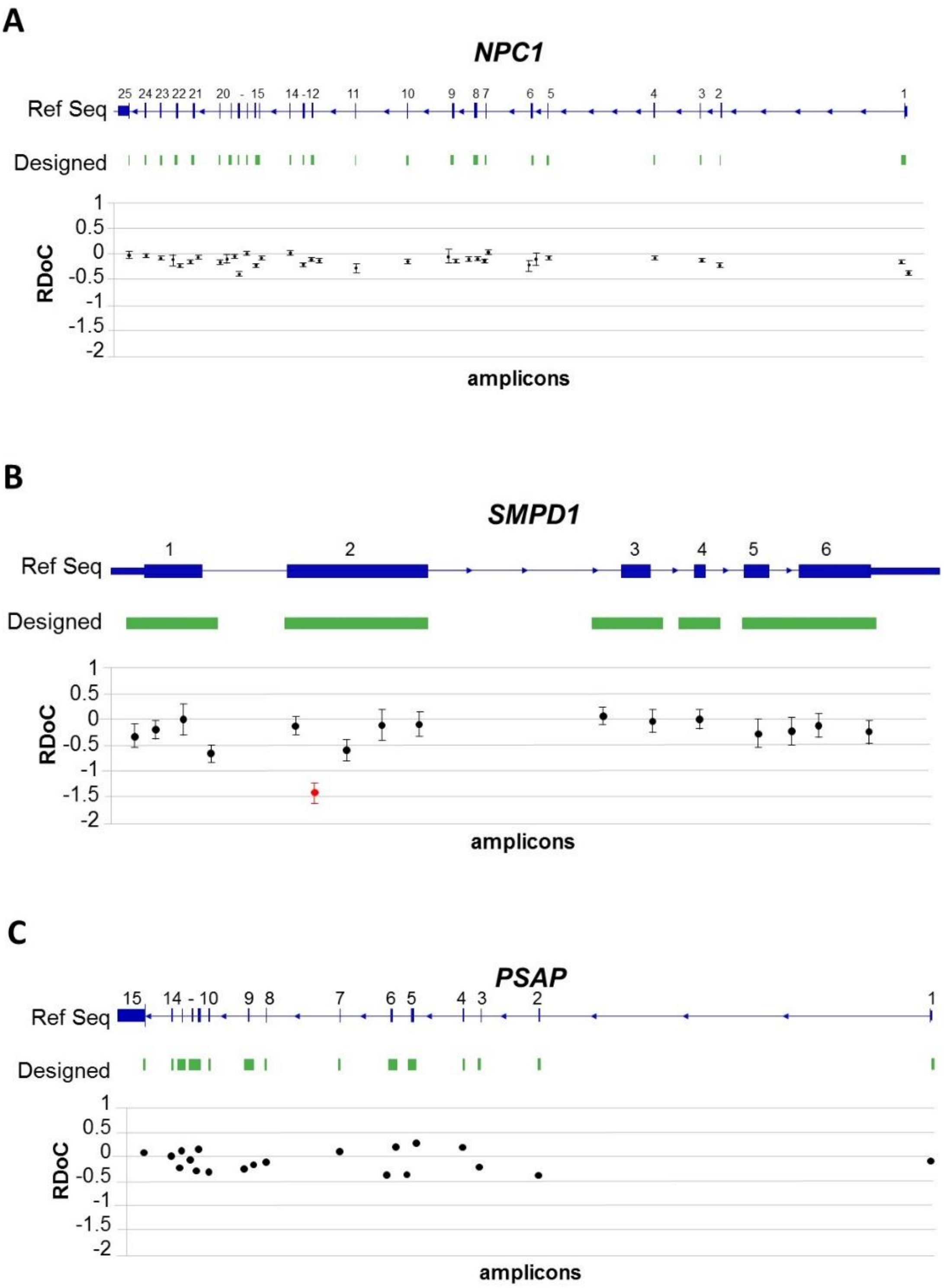

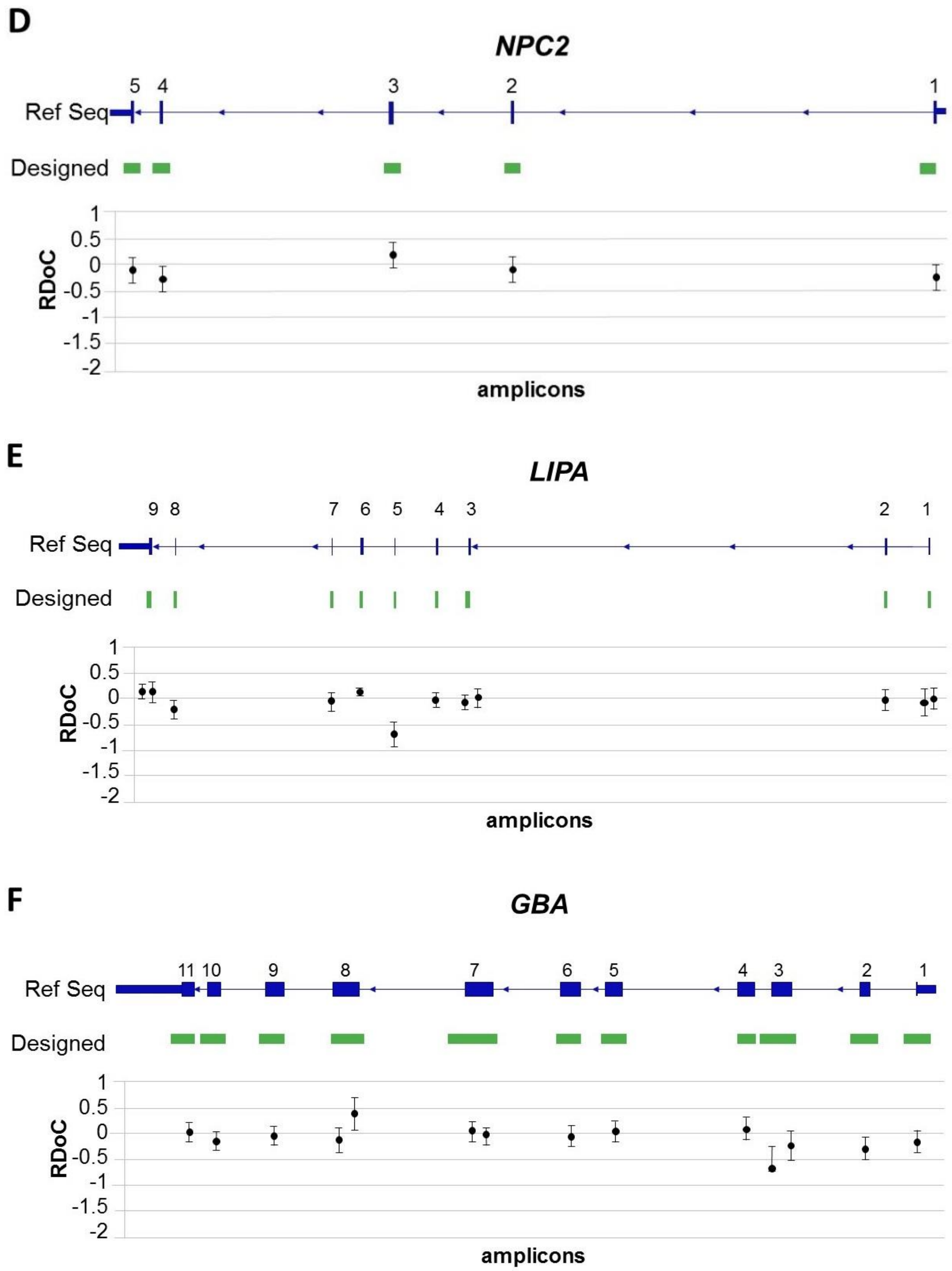
D
NPC2
5 4 3 2 1
Ref Seq
Designed
RDoC
1
0.5
0
-0.5
-1
-1.5
-2
amplicons
E
LIPA
9 8 7 6 5 4 3 2 1
Ref Seq
Designed
RDoC
1
0.5
0
-0.5
-1
-1.5
-2
amplicons
F
GBA
11 10 9 8 7 6 5 4 3 2 1
Ref Seq
Designed
RDoC
1
0.5
0
-0.5
-1
-1.5
-2
amplicons

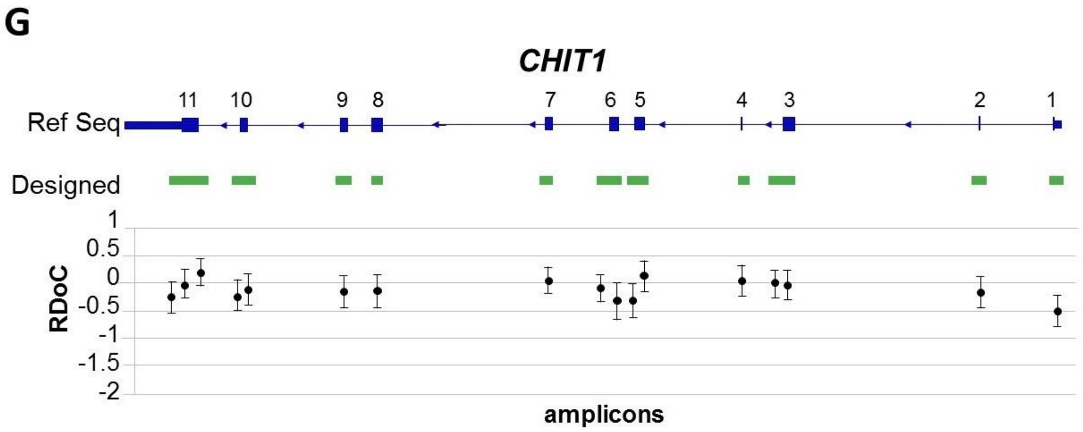

Figure S2 - Relative Depth of Coverage (RDoC) of 118 custom amplicons corresponding to eight-nine samples analyzed in one run. In blue is represented the reference sequence indicating the coding exons and in green the targets designed for NGS sequencing. A) *NPC1*, B) *SMPD1*, C) *PSAP*, D) *NPC2*, E) *LIPA*, F) *GBA* and G) *CHIT1*.
